# Understanding Patient Perspectives and Acceptability of Integrative Naturopathic Oncology in Metastatic Cancer

**DOI:** 10.3390/curroncol33100609

**Published:** 2026-10-10

**Authors:** Sara Izadi-Najafabadi, Adeleke Fowokan, Gurdev Parmar

**Affiliations:** 1 The Parmar Foundation, Surrey, BC V4A 1P6, Canada; research@theparmarfoundation.ca (S.I.-N.);; 2 Integrated Health Clinic, Surrey, BC V4A 1P6, Canada

**Keywords:** integrative oncology, naturopathic care, naturopathic oncology, metastatic cancer, acceptability

## Abstract

Individuals living with metastatic cancer frequently experience high symptom burden and reduced quality of life, leading many to seek integrative therapies alongside standard care. This study utilized the Theoretical Framework of Acceptability to systematically examine patient perspectives and the acceptability of integrative naturopathic oncology. Qualitative, semi-structured virtual interviews were conducted with 12 adult metastatic cancer patients who had received at least three months of care at a specialized Canadian clinic. The hybrid inductive-deductive analysis revealed high overall intervention acceptability. Patients reported positive emotional attitudes, strong alignment with personal values, improved treatment understanding, and increased confidence in managing their care. They particularly valued relational support, whole-person care, and an enhanced sense of agency. However, key implementation barriers included significant out-of-pocket expenses, social and family trade-offs, and navigating disconnected, uncoordinated healthcare systems. Integrative naturopathic oncology is highly acceptable to metastatic cancer patients as a valued complement to conventional regimens. To optimize equitable and safe care delivery, future health systems must reduce financial barriers and introduce structured communication channels between conventional and integrative health providers.

## Simple Summary

People living with metastatic cancer often seek complementary therapies to manage difficult symptoms and improve their overall well-being alongside standard treatments. This study explored how patients view and experience integrative naturopathic oncology care. Interviews revealed that patients find this care highly acceptable, valuing the emotional support, personalized guidance, and sense of control it provides during their cancer journey. However, individuals faced notable obstacles, including high out-of-pocket costs and difficulty coordinating care between disconnected healthcare providers. These findings highlight the need for healthcare systems to build stronger communication bridges between conventional cancer teams and naturopathic providers while addressing financial barriers. Ultimately, this research can inform future policies and clinical models to create more supportive, accessible, and coordinated care for people navigating advanced cancer.

## 1. Introduction

Cancer continues to pose a major global health burden, with an estimated 20 million new diagnoses and 9.7 million deaths recorded in 2022. These figures are projected to nearly double by 2050 due to aging populations, increasing incidence, and expanding mortality rates [1]. While progress in early detection and targeted treatments has led to improved survival, a substantial proportion of survivors are still diagnosed at metastatic stages [2], where they face a heightened risk of adverse outcomes and more treatment-related side effects, including pain, psychological distress and impacts to overall quality of life [3,4].

Integrative naturopathic oncology, which merges conventional care approaches with naturopathic oncology, has recently emerged as a treatment modality for addressing treatment-related effects of metastatic cancers, given its whole-body treatment approach. One systematic review exploring patterns of use of complementary and alternative medicine (CAM) in cancer patients across 18 countries found an overall prevalence of CAM in 40% of cancer patients, with the United States and Canada ranking high on the list of countries with the most CAM use. A growing survival rate among individuals with metastatic cancer underscores the importance of understanding their unique needs, as conventional clinical encounters often fail to capture the full range of symptoms, psychosocial concerns, and treatment priorities valued by patients [5,6].

Moreover, integrative naturopathic oncology is recommended but inconsistently implemented, in part due to mismatched system priorities and fragmented service delivery [7]. The dominant biomedical paradigm underpinning Western conventional medicine in North America has been slow to embrace integrative naturopathic oncology, partly due to educational gaps and perceptions of insufficient evidence [8,9]. Yet, despite systemic barriers to its uptake, evidence suggests that cancer survivors frequently seek integrative modalities to supplement standard care [10]. Exploring patient perspectives on the acceptability of integrative approaches can therefore support more person-centered and holistic care models, aligned with patients’ values/needs and conducive to better symptom management and overall quality of life.

Recent efforts by leading cancer bodies such as the American Society of Clinical Oncology (ASCO) and the National Comprehensive Cancer Network (NCCN) to endorse evidence-based complementary therapies—including meditation, acupuncture, and yoga—reflect a nascent shift toward bridging this divide and improving cancer care [11,12,13,14]. However, the leap towards the widespread acceptance and adoption of integrative naturopathic oncology, beyond foundational therapies like meditation and yoga, will require a robust evidentiary base. This underscores the timely need for integrative oncology researchers to generate high-quality evidence tailored to North American clinical contexts and contribute to the evolution of integrative naturopathic oncology for metastatic cancer survivors.

To this end, the present study utilized the Theoretical Framework of Acceptability (TFA) to systematically explore patients’ motivation for seeking integrative naturopathic oncology care and understand the acceptability of integrative naturopathic oncology care from the patient perspective.

## 2. Materials and Methods

### 2.1. Study Design & Setting

This study utilized a qualitative-descriptive design, as described by Sandelowski [15,16], to generate practical insights into patient perspectives and the acceptability of integrative naturopathic oncology in metastatic cancer. The study was reported in accordance with the Consolidated Criteria for Reporting Qualitative Research (COREQ) guidelines (see Supplementary Materials File S1) [17].

Substantial preliminary work was conducted with three volunteer patient partners to help inform the study design and ensure that patient priorities and lived experiences were meaningfully integrated into the research process. The patient partners included individuals living with a wide range of cancer diagnoses spanning early-stage disease to advanced metastatic conditions. Their input contributed to the refinement of the research questions and the development of the interview guide.

Subsequently, in-depth, semi-structured interviews, structured around the TFA constructs, were conducted with patients who had received integrative naturopathic oncology for metastatic cancer. The interview guide is provided in Appendix A. This study was conducted virtually with patients receiving integrative naturopathic oncology at the Integrated Health Clinic in British Columbia, Canada, which provides science-based naturopathic oncology care, between October and December 2025.

### 2.2. Recruitment

Participants were recruited through a newsletter invitation circulated to the clinic’s patient community, including current and former recipients of integrative naturopathic oncology. Interviews were conducted by S.I.-N., a female researcher with a PhD in Rehabilitation Science and training in occupational therapy, and extensive experience in qualitative health services research, including conducting semi-structured interviews and qualitative data analysis. Interviews were conducted virtually via Zoom (Version: 7.1.9; Zoom Communications, Inc., San Jose, CA, USA) and ranged from approximately 30 min to 1.5 h, with no non-participants present during the sessions. No repeat interviews were carried out.

Participants had an established clinical relationship with a GP (naturopathic doctor) and the Integrated Health Clinic team; however, no prior relationship existed between participants and the researchers before study recruitment. S.I.-N. sought to establish rapport through the consent process and by explaining the study objectives and procedures before the interviews and providing participants the opportunity to ask questions. Participants were informed that S.I.-N. was a researcher independent of their clinical care, and the research team maintained reflexivity throughout data collection and analysis by acknowledging and discussing potential assumptions during interpretation.

Eligible participants included adult patients aged 18 years or older who were initially diagnosed with metastatic cancer within the past 5 years, had received integrative naturopathic oncology for at least 3 months, and were able to communicate in English. Purposive sampling with maximum variation was employed to capture a range of experiences across factors such as age, sex, and cancer diagnosis.

### 2.3. Theoretical Framework

The TFA examines the extent to which individuals delivering or receiving a healthcare intervention consider it appropriate, based on anticipated or experienced cognitive and emotional responses. The TFA encompasses seven key constructs relevant to acceptability: affective attitude (patients’ feelings about the intervention), burden (perceived effort required to participate), perceived effectiveness (extent to which the intervention is seen to achieve its purpose), ethicality (alignment with personal values), intervention coherence (understanding of how the intervention works), opportunity costs (perceived benefits or opportunities forgone), and self-efficacy (confidence in engaging with required behaviors) [18].

### 2.4. Data Analysis

Data were analyzed concurrently with interviews using a hybrid inductive-deductive thematic analysis approach [19]. TFA was applied deductively to structure the analysis, while inductive open coding was conducted simultaneously to capture novel, emergent themes specific to patients’ experiences with integrative naturopathic oncology. Interviews were audio-recorded with consent and transcribed verbatim using the Fireflies.ai platform (Fireflies.ai Corp., San Francisco, CA, USA), verified for accuracy, and imported into ATLAS.ti platform (Version 24; ATLAS.ti Scientific Software Development GmbH, Berlin, Germany) for data organization and coding. Analysis proceeded through six iterative steps adapted from Fereday and Muir-Cochrane [19]: (1) development of an initial deductive codebook operationalizing the seven TFA domains; (2) independent dual-coding of an initial subset of five transcripts by two members of the research team (AF and SI) using a hybrid approach to capture both deductive framework domains and emergent inductive codes; AF is a male research associate with a PhD in public health and an experienced mixed-methods researcher. (3) codebook consolidation and refinement through iterative consensus; (4) mapping and synthesizing codes into overarching themes aligned with the TFA framework; (5) application of the finalized codebook to the remaining transcripts (by one reviewer) and synthesis of codes into overarching themes aligned with the TFA framework; (6) finalizing theme definitions and selecting illustrative quotes.

Data saturation was defined as the point at which no new codes, themes, or insights emerge from the data and was reached early in the analysis based on the final iteration of the codebook [20]. The initial transcript (Participant 1) established the core codebook, comprising 25 codes. Participant 2 introduced four additional codes (Opportunity Costs: Manageable side effects; Self-efficacy: Low competency; Motivation: Social influence and peer validation; and Perceived Effectiveness: Perceived lack of benefit), and Participant 3 introduced the 30th and final unique code (Burden: Social and relational strains). From Participant 4 onward, code stability was achieved, with no further unique codes emerging across the remaining nine interviews (Participants 5 through 12).

Theoretical saturation was defined as the point at which the full range of constructs comprising the underlying framework is comprehensively represented [20]. Because the TFA was applied a priori to structure the interview guide, it was likewise achieved early in data collection. Interviews were nevertheless continued through a total of 12 participants to ensure analytical depth, confirm thematic stability across diverse participant experiences, and verify that both data and theoretical saturation were fully maintained.

Cross-cutting modality-specific patterns were also identified, describing how acceptability experiences varied across different integrative therapies; these patterns are presented separately and were not treated as an additional overarching theme.

### 2.5. Trustworthiness and Rigour

Trustworthiness was addressed through credibility, dependability, confirmability, and transferability. Credibility was strengthened through purposive maximum-variation sampling, audio-recording and verification of verbatim transcripts, and independent dual coding of five transcripts. Coding discrepancies were resolved through discussion, and the codebook was refined iteratively. A hybrid deductive–inductive approach enabled analysis to be guided by the TFA while retaining participant-generated experiences beyond the framework. Data collection and analysis occurred concurrently, code stability was monitored, and illustrative quotations supported links between the data and reported themes.

Member reflection was undertaken after manuscript development. A patient partner who had contributed to developing the research question reviewed the manuscript, particularly Section 3, and commented on the resonance, balance, clarity, and faithfulness of the interpretations. Their feedback affirmed the representation of both positive and negative experiences.

Dependability was supported through a documented analytic process, including codebook development, independent coding, consensus-based refinement, and application of the final codebook to remaining transcripts. Coding decisions, codebook iterations, and theme development were retained in Atlas.ti and study documentation.

Confirmability was enhanced through reflexive discussions of researchers’ assumptions, consensus-based interpretation, and use of verbatim quotations. Interviews and analysis were conducted by researchers independent of participants’ clinical care, helping to mitigate potential influence of clinic-based relationships and the clinical co-author’s role.

Transferability was supported through detailed reporting of the setting, participants, recruitment, care received, interview procedures, and analysis, enabling readers to judge relevance to other metastatic cancer and integrative oncology contexts.

### 2.6. Ethics

Ethics approval was received from the Canadian College of Naturopathic Medicine Research Ethics Board (CCNMREB065.Parmar). Informed consent was obtained from all individual participants included in the study prior to data collection.

## 3. Results

### 3.1. Participants

A total of 63 individuals expressed interest by completing the screening form following the newsletter invitation. Of these, 42 were excluded because they did not meet the eligibility criteria. Specifically, exclusions were due to the absence of an integrative oncology treatment history (*n* = 10), lack of metastatic cancer diagnosis (*n* = 10), missing data (*n* = 10), diagnosis or treatment occurring more than 5 years prior (*n* = 9), duplicate submissions (*n* = 2), or identification as a patient partner (*n* = 1). The remaining 21 individuals were contacted to review the consent form and receive additional information about the study. Of these, five did not respond to follow-up contact. Among the 16 individuals who provided consent, two participants missed their scheduled interview appointments, and two became unresponsive thereafter.

Twelve patients who had received integrative care for at least three months were interviewed (10 females, 2 males; age range 18–79). Cancer types included colorectal, breast, lung, and other cancers. Table 1 provides an overview of the characteristics of study participants.

Participants reported receiving a range of integrative naturopathic oncology therapies including intravenous therapies such as high-dose vitamin C and mistletoe injections, acupuncture, massage, craniosacral therapy, nutritional therapy, and hyperthermia. The duration of care varied from a few months to over three years, with many participants engaging in multimodal treatment approaches over extended periods.

### 3.2. Themes

A total of 30 unique initial codes were extracted across all interviews. Because the primary study objective was to understand the acceptability of integrative naturopathic oncology, 26 codes were classified under the seven deductive domains of the TFA: Affective Attitude, Burden, Ethicality, Intervention Coherence, Opportunity Costs, Perceived Effectiveness, and Self-Efficacy. The remaining 4 codes captured patient-driven drivers that transcended the temporal scope of intervention acceptability and were inductively categorized into an eighth emergent category: “Motivations for Engaging in Integrative Naturopathic Oncology.” Within each category, initial codes were iteratively grouped into functional subcategories based on thematic similarity before synthesizing final qualitative themes. Table 2 provides an overview of the thematic findings mapped to the seven TFA constructs, the emergent motivations theme, and cross-cutting modality-specific patterns.

#### 3.2.1. Affective Attitude

**Relationally positive but systemically constrained care experiences**: Participants generally described integrative naturopathic oncology care in positive relational terms, emphasizing feelings of emotional support, trust, and being genuinely cared for as individuals rather than “just another patient.” Many valued the personalized and holistic nature of the care, as well as the sense that providers were invested in their overall well-being and accessible throughout their cancer journey. Participants frequently contrasted these experiences with more impersonal or task-oriented interactions encountered within conventional oncology settings. Participant#8, a woman in her 40s diagnosed with colorectal cancer said:

*“I can’t even tell you how different it was now and still is. Like, I still go through this with both my oncologist and, you know, Dr (integrative oncologist. it’s incredible. It’s like talking to somebody that I trust in the integrative side, that I know cares about me, that has my back, that is invested in this and, give me all the support that I need, rather than somebody that’s like, I’m just a number and just another case that they’re dealing with and, that they don’t really care about.”*

However, alongside these positive experiences, some participants expressed frustration with the marginalization of integrative care within the broader cancer system and feeling caught between two systems of care. For some, these structural constraints contributed to disappointment. Participant#11, a woman in her 50s diagnosed with breast cancer, mentioned:

*“I would say that actually I’ve been pretty disappointed with integrative care. And the reason why is because I want it to be, I’ve wanted it to be my primary source of care. And I feel like I don’t know how things work in the industry, but it’s almost, it’s always just considered to just be this complementary. I don’t like the word complementary, but it’s approached as complementary. I also feel conventional oncologists are in charge. This is the order of operation and then whatever they say is what goes. And then integrative or naturopathic care is somehow below that. You as a person are the patient, are left in the middle trying to defend yourself. And there’s very poor communication.”*

*“And both of them said, you have to do chemo. And my (integrative oncologist) specifically, I was like, I need you to look me in the eye. Like. Like, I don’t feel like this is the right choice for me. And he’s like, you have to do it. And I remember reading something after I left his office at some point saying that seeing that naturopathic physicians could not counteract or contradict convention, if a conventional oncologist has recommended chemo, they have to go along with that recommendation, too. And I remember feeling so betrayed.”*

**Participant#11**

**Evolving perception of integrative naturopathic oncology**: Participants described differing trajectories in their attitudes toward integrative naturopathic oncology following their metastatic cancer diagnosis. Some participants who had previously been skeptical of integrative approaches and oriented toward conventional evidence-based treatment described becoming more open to integrative care after confronting the limitations of conventional treatment and the realities of advanced or terminal illness. This shift reflected a broader re-evaluation of what constituted meaningful treatment, particularly when cure or substantial survival gains were uncertain. Participant#2, a woman in her 40s diagnosed with lung cancer, said:

*“So if you would have asked my pre cancer diagnosis self about going down this path of integrated care, I probably would have laughed at myself, to be completely honest and said, save your money, stick with what we have, you know, randomized clinical trials on and you know, medications that have been shown to be effective. But then you’re faced with the realization that the current state of medications that we have, even though they’re effective in randomized clinical trials, you know, sometimes that effectiveness means two months longer of living. So it’s not very. You know, on paper that looks great, but when you’re faced with that reality, you realize that those benefits maybe aren’t as good as you would have wanted them to be.”*

In contrast, other participants approached their cancer experience with pre-existing beliefs about naturopathic, holistic, or integrative health. For these individuals, the diagnosis did not alter their philosophy, but rather reinforced it, prompting them to seek specialized integrative naturopathic oncology care and strictly adhere to treatment recommendations.

#### 3.2.2. Burden

**The multidimensional burden of integrative care:** Participants described a multidimensional burden associated with integrative naturopathic oncology, encompassing interpersonal, physical, emotional, and financial domains. At the interpersonal level, some participants experienced tension within their social and familial networks, particularly when their decision to pursue integrative approaches was questioned or not supported by others. These reactions at times intensified emotional distress and reduced confidence in their treatment choices. Participant#3, a woman in her 40s diagnosed with breast cancer, said:

*“And then friends, you know, the friends and my husband are going like why you would decide to do that [integrative oncology]?”*

In addition to social strain, participants reported that integrative treatments themselves could be physically and emotionally demanding, with some procedures described as difficult to tolerate and contributing to mental and physical exhaustion. Participant#1, a young woman in her 20s with lung cancer metastasized to liver, said:

*“There were times where I had found treatments hard, like doing my fevers, getting induced in fevers. It’s hard mentally and physically.”*

Financial and access-related constraints further compounded this burden, with participants describing the prohibitive cost of therapies, lack of insurance coverage, and logistical hurdles such as travel as significant barriers. Together, these overlapping demands created a cumulative strain, forcing participants to constantly balance emotional, physical, and financial resources while navigating their care.

*“Like for me that’s the biggest thing is I just can’t afford all of the treatments, and I know the treatments could be huge to prolonging my life and you know, it’s something that I don’t have that option of. So that’s the hard part.”*

**Participant#4, a man in her 40s diagnosed with colorectal cancer**

*“I’d say the most difficult is finances because no insurance really covers the supplemental therapies that you tend to get … So I don’t see it as an unnecessary expense. But often when you’re doing six things you don’t quite know are all six working? You know, could I drop one and save some money? You know, especially as the sole breadwinner for a family with six kids, a wife and six kids. You know, you’re constantly trying to balance how much is going out. On that as far as the energy and emotional.”*

**Participant#6, a woman in her 50s diagnosed with colorectal cancer**

**Navigating fragmented systems of care and contested legitimacy:** Participants described integrative naturopathic oncology care as occurring within a deeply siloed healthcare environment where conventional oncology and integrative naturopathic oncology had differing beliefs and often operated in parallel rather than in seamless collaboration. Communication between providers was typically indirect, leaving participants feeling that the two approaches were not well aligned. Some participants also felt compelled to withhold information about their use of integrative therapies due to concern that disclosure might negatively influence their conventional treatment plans, reinforcing a sense of needing to navigate between two fragmented systems.

*“For the most part, I liked everything. And I don’t know if this is a problem with integrative oncology, but I struggled with bringing the two sides together. Like kind of the medical oncology with the integrative oncology! they don’t talk.”*

**Participant#7, *a woman in her 50s diagnosed with ovarian and breast cancer***

*“I think it’s terrible that I have to hide a lot of the things that I’m doing for my oncologist for fear that she’ll not want to give me my chemo or proceed with treatments because she doesn’t agree with what I’m doing”*

**Participant#8, a woman in her 40s diagnosed with colorectal cancer**

In contrast, **Participant#12, a woman in her 60s diagnosed with ovarian cancer,** initially felt caught between the two approaches but later developed confidence in navigating both and making her own informed treatment decisions. She mentioned:

*“So I don’t feel now that there’s a division, I feel like I’m actually working quite well with both. I mean, at the beginning. It was definitely hard at the beginning because I wasn’t sure, you know, I didn’t want him. You know, I didn’t want to upset one and I didn’t want to upset the other. But, now it’s like, no, this is my decision and, you know, give me the facts, I’ll do my research, but ultimately it’s my decision. So, I don’t feel that pull anymore.”*

Alongside these structural gaps, participants described uncertainty around the legitimacy and visibility of integrative naturopathic oncology. Some were initially unaware that specialized integrative oncology services existed, while others noted that integrative approaches were sometimes viewed with skepticism or confused with alternative therapies. This lack of clarity and recognition contributed to hesitancy or delayed engagement for some participants. In addition, perceived dismissiveness or lack of endorsement from conventional clinicians appeared to shape how much trust participants placed in integrative naturopathic oncology, further reinforcing tensions around legitimacy and integration within the broader cancer care system.

*“I don’t think it’s visible enough, at least the cancer community. And I do think it has a bad reputation, or I think it’s getting better, but a lot of people are very wary of it, I should say. So. There are some people in the community who are very anti. Or they get. I should say they sometimes, I think, get confused with integrative oncology and alternative treatments.”*

**Participant#7**

#### 3.2.3. Opportunity Cost

**Accepting hardship in pursuit of potential benefit**: Participants described a range of sacrifices and trade-offs that accompanied engagement in integrative naturopathic oncology care. Despite enduring physical, emotional and logistical challenges, many framed these hardships as acceptable compromises when balanced against perceived health benefits, symptom management, or hope for improved well-being. Chief among these hardships were family and relational trade-offs which were commonly described when treatment required travel, time away from home, and reliance on others for caregiving responsibilities. Participants frequently experienced a profound emotional toll rooted in temporary disconnection from their support systems. This isolation fostered mixed feelings where participants felt guilt over burdening their loved ones despite a steadfast belief in the value of the treatment.

*“I’d take time away from my family and from my support system. I often would be alone, traveling, because I mean, you know, obviously couldn’t afford to, you know, have my husband off work and traveling at the same time. I have two little boys, so, you know, you need somebody at home with them, so you. You feel alone. And I think that’s. That’s tough as well because you’re, you know, you don’t have your community behind you. So that felt a little lonely. So, you know, didn’t love that.”*

**Participant#8**

Beyond interpersonal strains, participants reported lifestyle, financial and physical sacrifices which included leaving work, reducing leisure activities, and enduring treatment-related discomforts such as side effects or difficulty tolerating specific supplements and procedures. While these disruptions were sometimes experienced by participants as significant losses, they were typically framed as temporary and manageable hurdles.

*“Yeah, I had to give up, I mean a lot of things like things that weren’t needs in order to pay for my care there. I had to give up all the time and I had to leave work and so things like that. I had to leave my family, my home. But it was one of those things where it’s like I’m not leaving forever and I had to do what I had to do. So it wasn’t scary being there. It was always relaxing. Actually. I enjoyed going in for the hour or two a day and like getting after. You get to know the nurses and talking with everyone and so you give a little, but you also get a lot back.”*

**Participant#1**

#### 3.2.4. Perceived Effectiveness

**Dynamic appraisal of integrative naturopathic oncology effectiveness**: Participants engaged in an ongoing evaluation of their integrative naturopathic oncology care, with perceptions shifting over time from initial uncertainty, disappointment, or skepticism to experiences of meaningful and often unanticipated benefit. Some participants ultimately described the care received as exceeding initial expectations, offering energizing or restorative effects that sometimes stood in stark, positive contrast to the grueling impact of conventional treatments:

*“I was kind of in a shock state, not really knowing what to expect… and to be super honest, it has exceeded everything that I expected.”*

**Participant#4**

*“I always find it more building than depleting going in for integrative oncology, you don’t, you know, when I walk out of chemo, I feel like I did chemo. When you walk out of, you know, doing hypothermia or vitamin C, you feel better. Like at least I do, you know, hyperbaric oxygen and same like you feel more energized.”*

**Participant#6**

However, a few participants experienced limited or no perceived benefit. This was particularly evident when symptoms worsened early in treatment, leading to disappointment and doubt:

*“I was so sick during that time and just getting worse that I think I, you know, I felt that maybe it wasn’t providing any additional benefit then, you know, nothing seemed to be working. … I just seemed to be getting worse. And so, at first I was a little bit disheartened by that.”*

**Participant#2**

#### 3.2.5. Ethicality

**Ethical alignment shaped by prior beliefs and lived experiences**: Participants’ ethical alignment with integrative naturopathic oncology was rooted in pre-existing beliefs, prior experiences with complementary or traditional healing practices, and broader conceptualizations of health and wellness. For individuals with a longstanding engagement with naturopathic care or culturally rooted healing approaches (e.g., indigenous ways of knowing), integrative naturopathic oncology was perceived as inherently credible, familiar, and trustworthy.

*“I’m always interested in nutrition and superfoods and taking care of my body and fitness and health… So I’ve always worked with naturopaths… So I knew right away that I wanted, you know, something in addition to Western medicine.”*

**Participant#8**

Ethical alignment was further reinforced through experiences of accessible, responsive, and supportive care. Participants emphasized the importance of being able to easily contact providers, receive timely and conscientious responses to questions and feel understood throughout their treatment experience. Ultimately, this responsiveness contributed to a profound sense of safety, trust, and reassurance, directly reflecting participants’ expectations of patient-centred care within integrative oncology.

*“So anytime I need to, like, call or anytime I have a question… I know I can pick up the phone or I can book a video call and just get something out of the way if I’m worried about it.”*

**Participant#1**

**Preferred models of care within integrative naturopathic oncology**: Participants expressed a clear preference for care delivery structured around multiple, overlapping health domains including integrative, naturalistic, and holistic patient-centred approaches that collectively reflected their broader expectations of “good” cancer care. Specifically, most participants endorsed models that explicitly integrated conventional oncology with complementary integrative therapies in ways that were perceived as supportive, holistic, and person-centred. Within this framework, integrative oncology was understood as augmentative rather than alternative. Participants valued complementary therapies because they mitigated side effects of conventional regimens and enhanced overall well-being alongside standard cancer treatments.

*“And, and it’s really important for me that I have traditional [natural indigenous] medicine on board as well as that, the mental and spiritual part that comes with that, as well as the, you know, what was being provided through BC Cancer.”*

**Participant#4**

*“My integrative oncologist looking at 15 things and you’re looking at two. But so that also has led to much more personalized care because you’re treating the person holistically and you’re tracking a much broader set of information so you can tweak things when necessary. Rather than just following the protocol that’s outlined at the beginning.”*

**Participant#6**

*“I don’t want to do anything that would compromise the medication that I’m taking… They’re not there to block the treatments that are also offered through BC Cancer, but to augment them and to make them more effective.”*

**Participant#2**

#### 3.2.6. Intervention Coherence

**Developing understanding through research, experience, and consultation**: Participants built their conceptual understanding of integrative care through a combination of in-depth conversations with clinicians, prior experiences, and independent research. Integrative care providers played a vital role in facilitating this health literacy, supporting participants to make informed treatment decisions tailored to their unique needs. Rather than viewing them as conflicting, most participants recognized that integrative therapies were designed to support, rather than interfere with conventional regimens. Importantly, participants made sense of integrative treatments by linking them to intrinsic biological processes. For example, understanding bodily processes such as fever or immune responses allowed participants to comprehend the physiological basis for treatments like hyperthermia, ultimately increasing their confidence in the utility of these therapies in managing their symptoms.

*“Really well. Yeah. So, I ask a lot of questions. I’m very interested in the medical field. I don’t know, it’s just something I’ve always been interested. Like I mentioned before, like, health and wellness have always been, you know, a passion of mine. So, understanding treatments and what it does to your body! I researched everything. I’d always look it up if I’m doing this [treatment]! Where did this come from? Where was it started? What are the outcomes of it? I’d look at studies that were out there. I think it’s important to understand what it’s doing to you. Especially when you’re paying for something, you know, you don’t want to just throw money at the wall and flush down the toilet.”*

**Participant#8**

*“My wife jokes around that I have a PhD in that treatment now. I understand them pretty well and the different mechanisms at play. And I wouldn’t say I understand cancer because I don’t know if anybody can understand it. I don’t know why some things work for some people really well and not for others. But I understand the methods of action of what I’m doing and how with certain treatments you get a cumulative effect like IV vitamin C, hyperthermia and hyperbaric oxygen … And then I feel like integrative doctors do a much better job explaining methods of action and why, you know, you’re doing things.”*

**Participant#6**

#### 3.2.7. Self-Efficacy

**Building confidence through knowledge and guidance**: Patients described that their confidence in managing treatment was shaped by increased knowledge and clear guidance from their care providers. Many emphasized that understanding their condition and treatment options helped reduce uncertainty and supported more confident decision-making. Some patients also actively sought additional information through personal research or second opinions, which further reinforced their confidence and reassurance.

Importantly, provider communication played a key role in this process, as step-by-step explanations and structured treatment plans helped patients feel less overwhelmed and more capable of engaging with complex integrative protocols. Together, these experiences suggest that self-efficacy was strengthened through both knowledge acquisition and supportive clinical relationships.

*“Just I guess on research that I had done myself and just kind of knowing that if we take care of ourselves, we can heal. And so why not try that first before dumping a bunch of chemicals and possibly having organ failure? That’s kind of what it came down to. When I first was diagnosed, I was told that I needed a liver transplant, like yesterday. I still haven’t received one and I’m fine. So kind of slowing down and just taking the time to get second, third opinions and taking your own, like getting your own aspects of everything and making your own decisions is important. And I think, yeah, after doing that and then after I talked to (integrative oncologist), I felt like I did the right thing.”*

**Participant#1**

*“But any questions I had were always answered and everything was explained to me before going in and nothing was forced on me either. It was all like, my choice, of course, to do any of this.”*

**Participant#1**

*“Yeah, I mean, there’s so much information. It does become a bit overwhelming sometimes. But one of the things that Dr. (integrative oncologist) had said to me at the very beginning, and I try and keep this in my mind is you don’t have to do everything all at once. She writes impeccable notes for me, puts things in step by step, has everything there, the office has everything. So, if I’m confused about anything, I can phone. There is so much information. So, it does become overwhelming with that. However, and it is time consuming. Like, you know, getting everything organized and taking your supplements when you’re supposed to be taking them and just being really organized with it has been very helpful.”*

**Participant#4**

**Taking an active role in cancer care**: Many participants demonstrated a commitment to active engagement across their treatment trajectory, reflecting increased self-efficacy in managing their care. Rather than adopting a passive stance after their diagnosis, participants emphasized a deliberate commitment to adhering to clinical recommendations and seamlessly integrating care protocols into daily routines. Importantly, when faced with notable challenges such as logistical complexity or systemic fatigue, participants characterized these challenges not as barriers but as manageable disruptions, with participants viewing their involvement as an indispensable and meaningful component of their broader cancer care.

*“Wasn’t too hard. You know, sometimes a lot of the different supplements can be confusing, you know, and when to take them and how to take it and you know, A little bit overwhelming sometimes. So that, you know, can be a thing. But, you know, like I mentioned, I. I love that. That kind of stuff. So to me, you know, going and like, sourcing out, where to buy them, what to do and, like, you know, preparing my routine for the day and all the different things I need to take and do. I don’t mind doing that. And I’m very, you know, good at that routine and those reminders. So that works for me. You know, probably it would be more difficult for. For others and maybe a little overwhelming with all the different things.”*

**Participant#8**

*“it’s not sustainable for everybody, but it, I’m able, I’ve been able to do it. Like, some days it’s a struggle, but it is what it is, and I think that it does help and it will help me in the long run as well, even if I didn’t have a diagnosis.”*

**Participant#1**

**From patient to advocate**: For some participants, increased self-efficacy in integrative oncology extended beyond their personal care management and evolved into active advocacy for others. Participants described purposefully sharing their experiences with friends, family members, and coworkers facing similar diagnoses, often encouraging them to explore integrative oncology options or seek specialized clinical expertise before finalizing treatment decisions. This willingness to speak openly about their care reflected deep trust in the integrative modality and a profound sense of responsibility to support peers navigating similar diagnoses and prognostic realities. Overall, these narratives suggest that self-efficacy in integrative naturopathic oncology contexts may extend beyond individual treatment management to include a broader commitment to fostering patient education and peer support.

*“they’ve seen me go through this and they see me be successful. So, you know, I, I am, you know, always sharing my knowledge with them and the information that I’ve learned. And I believe that an integrative approach is incredibly important.”*

**Participant#8**

*“And then once I learned about it, then I shared with you other people because there’s two other people at work that got cancer, actually three and I. And the one guy did go, but he wasn’t, he decided not to go with integrated.”*

**Participant#9, a man in his 60s diagnosed with oral cancer**

#### 3.2.8. Motivations for Engaging in Integrative Naturopathic Oncology

**Seeking relational care and emotional reassurance**: Participants were motivated to engage in integrative naturopathic oncology by a desire for relational care and emotional reassurance they often found lacking in conventional oncology settings. Many participants valued providers who practiced active listening, explained things clearly, and maintained an accessible, warm and caring presence, which helped them feel seen as whole persons rather than as cases or numbers. Conversely, conventional care was sometimes experienced as brief, fragmented, and task-oriented, forcing participants to continuously solicit relevant information or repeat their concerns, which routinely left them feeling unheard or unsupported. In this context, integrative oncology was appealing not only for its therapeutic benefit, but because it cultivated a more personal, trusting, and sustaining relationship that provided comfort across the treatment trajectory.

*“I just wanted individual attention. I guess I didn’t want to go to a group and with other people because I don’t really like that kind of thing. But I just wanted somebody to talk to at the same time that had a different take to what I was going through. So that. And once I found that place that was like, I’d been telling everybody about it.”*

**Participant#10, a woman in her 50s diagnosed with colorectal cancer**

*“Dr. (integrative oncologist) especially is really good at, you know, encouraging and you know you’re not a number, you’re not a percentage. You’re patient, and it’s more about how you’re doing. So he’s really good at that.”*

**Participant#10**

*“Night and day, like, incredibly different. I can’t even like my oncologist. I think she has zero bedside manner. She is not. She’s not supportive. She doesn’t celebrate your wins. I never felt. I never felt good. I guess it was more like very clinical. Very, like, you know, looking at my chart as she’s talking to me. She doesn’t know who I am. She wouldn’t remember anything about my background. She asked me the same questions over and over.”*

**Participant#8**

**Seeking agency and hope in the face of uncertainty**: Participants were also motivated by a desire to reclaim agency and preserve hope in the face of uncertainty. When conventional treatment felt limited or difficult to navigate, integrative naturopathic oncology offered participants a tangible way to take action, restoring a sense of control over an otherwise unpredictable illness experience. The proactive pursuit of additional options afforded participants a sense of agency, enabling them to actively contribute to their own care rather than being passive recipients who were simply enduring their diagnoses. For some, this motivation was tied to preserving hope and extending life, even despite poor prognosis, with integrative care serving as a tool for personal determination, where participants could continuously exert choices and influence the course of their treatment journey.

*“Well, firstly, because I was scared, you know. And because I had been diagnosed at such a late stage, four, because it had already metastasized, it metastasized into my peritoneum. My peritoneum as well as my liver. And, and because … I Had a CT scan that showed just a light shadow on my liver, and that was in April of 2022. By June, when I actually went in again to the hospital and they did another CT scan, my liver was riddled with cancer. So in two months, yeah, two months it had progressed to that. So, you know, very scary.”*

**Participant#12**

*“I felt like I was doing something. Felt like I was doing something for myself rather than, like, feeling weak and like, this is happening to me. Like, it’s happening, you know, I’m making it happen as well. Yeah.”*

**Participant#8**

*“I guess I felt kind of that I didn’t have anything to lose by doing more than what the cancer agency was offering me. Just knowing that I didn’t have a very good prognosis and just wanting to give myself as much time as I could.”*

**Participant#2**

Seeking validation through peer’ experiences: Some participants’ decision to engage with integrative care was motivated by the positive experiences of coworkers, friends, or other participants who had previously used integrative naturopathic oncology. Their testimonials played a vital role in normalizing the integrative care modality, mitigating concerns that might be associated with navigating a new therapeutic landscape.

*“One of my coworkers … had known one of the nurses … who had gone through Integrated Health Clinic… She shared her story with me. And that’s how I initially had thought.”*

**Participant#2**

*“I have a friend who’s been seeing a naturopath … My friend just mentioned, okay, just seek these doctors because they’re more of finding or treating the integrative way.”*

**Participant#5, a woman in her 70s diagnosed with endometrial cancer**

### 3.3. Cross-Cutting Modality-Specific Patterns

While participants engaged in diverse integrative oncology regimens, distinct qualitative patterns emerged across specific therapeutic modalities:

- **Intravenous (IV) Vitamin C** was discussed in relation to *Perceived Worth & Benefits* (*n =* 5), with participants often reporting immediate post-infusion improvements in energy, appetite, or well-being. Concurrently, it was the primary modality associated with *Gaps in Conventional-Integrative Collaboration* (*n* = 4), as patients frequently disclosed or debated its use with their conventional oncology teams.
- **Hyperthermia** was characterized by higher frequencies of *Treatment-Related Burden* (*n* = 2) and *Perceived Lack of Benefit* (*n* = 3). Participants described the physical intensity and discomfort of hyperthermia protocols (both local-regional and whole-body).
- **Mistletoe Therapy** was associated with *Knowledge-Building through Research & Consultation* (*n* = 3), *Perceived Worth & Benefits* (*n* = 3), *manageable side-effects* (*n* = 3) and *Financial/Access Burdens* (*n* = 3). Patients noted requiring substantial personal research and consultation to understand its rationale, contrasting with more familiar natural health concepts.
- **Intervention Coherence & Modality Familiarity**: Concepts of Intervention Coherence varied by therapy type. Modalities like Vitamin C (*n* = 3) and Hyperthermia (*n* = 2) were readily linked to biological mechanisms and bodily processes (e.g., nutritional/antioxidant support and heat/fever response, respectively), whereas Mistletoe (*n* = 4) required proactive research and consultation to achieve coherence.

**Participant#2 said:** “Yeah, so the hyperthermia I didn’t do a whole lot of research on because I felt that it had Very minimal chance of interacting with my treatment. You know, like, in my head, it wasn’t. It didn’t raise red flags about being harmful in any way or harming my treatment. The mistletoe and the vitamin C, I definitely looked into kind of on two different fronts. One, to make sure that they didn’t interact with my medication, and two, that it didn’t have any impact on my liver failure symptoms because many natural health products, you know, have been known to have side liver. And so I wanted to make sure that nothing would be harmful for my liver either.”

## 4. Discussion

Overall, participants described integrative care as acceptable because it aligned with their values, fostered trusting therapeutic relationships, and supported a sense of agency in the context of advanced disease. While integrative oncology approaches are widely utilized across Canada and the USA, their integration into mainstream cancer care remains limited due to the dominant Western biomedical paradigm and insufficient evidence [8,9]. This qualitative study represents an important first step in understanding the acceptability of integrative oncology interventions among cancer patients. By demonstrating that patients are willing to engage with and perceive value in integrative approaches collaboratively with conventional medicine, this research provides preliminary evidence that structural, physical and financial barriers may be more significant than patient acceptance barriers. These findings suggest that further research, particularly quantitative randomized controlled trials examining effectiveness, is needed to complement acceptability evidence and support organizational recommendations for integrative oncology integration into standard cancer care protocols.

This qualitative descriptive study used the TFA to explore how patients with metastatic cancer experienced integrative naturopathic oncology. Across the seven TFA domains, participants’ responses showed acceptability across multiple dimensions, with ethicality and effectiveness emerging as particularly salient themes. Ethicality was central to participants’ acceptance of integrative approaches. Many participants entered care with prior belief or experience of naturopathic or traditional healing, or with a preference for treatment approaches that complement rather than replace conventional oncology. Most viewed integrative oncology as a way to support standard cancer therapy by mitigating side effects and improving overall well-being. Effectiveness, while more nuanced, reflected participants’ perceptions of symptom management, quality of life improvements, and the synergistic potential of combined conventional and integrative approaches. These findings suggest that acceptability of integrative oncology is fundamentally rooted in alignment with patient values and perceived clinical utility, rather than being driven solely by biomedical efficacy evidence.

While participants often demonstrated high self-efficacy and a willingness to tolerate treatment burdens in pursuit of perceived benefit, this commitment came at considerable cost (aka. opportunity cost). Participants described tension within families and social networks, fatigue related to maintaining multiple treatments, and substantial out-of-pocket expenses. These burdens were particularly salient in the Canadian context, where many integrative modalities remain privately funded and are therefore accessible only to those with sufficient financial resources and logistical flexibility. Participants’ perceptions of treatment effectiveness evolved over time. Some described a shift from relying solely on conventional therapies toward embracing integrative oncology, reflecting a desire to incorporate the “best of both worlds” by combining conventional and complementary approaches. Clinician explanations of mechanisms and expected effects appeared to strengthen intervention coherence and trust, supporting more active and informed participation in care.

Participants consistently emphasized the importance of person-centred, relational care, illustrating the affective attitude domain of acceptability. Integrative encounters were described as unhurried, attentive, and responsive to emotional as well as physical needs, contrasting with experiences of brief and task-focused conventional oncology visits. These findings are consistent with prior qualitative research showing that people with advanced cancer value holistic care that addresses psychosocial well-being alongside disease-directed treatment [21,22]. At the same time, participants’ accounts pointed to fragmentation between conventional oncology and integrative naturopathic oncology. Limited communication between providers often required participants to coordinate care themselves, and some felt reluctant to disclose integrative therapy use because of concerns about judgment or perceived conflict with standard treatment. This communicative gap underscores a persistent tension in the integrative oncology literature, where institutional skepticism frequently compels patients to navigate their supportive care choices in silence [23,24,25,26].

Moreover, while this finding highlights a strength of integrative approaches, it is important to recognize that person-centered care is not and should not be unique to integrative medicine. Rather, these findings suggest that the person-centered methodologies employed within integrative settings offer an instructive model for conventional oncology. The recognition that cancer patients require comprehensive attention to their psychosocial, emotional, and physical wellbeing is not incompatible with evidence-based conventional treatment. Instead, the integration of person-centered care principles into conventional oncology settings represents an opportunity to enhance patient experiences while maintaining rigorous scientific standards. This dual commitment—to both evidence-based medicine and person-centered care may ultimately facilitate greater acceptance and uptake of integrative approaches within mainstream cancer care.

Our findings highlight that the acceptability of integrative naturopathic oncology is not uniform; rather, acceptability is heavily shaped by the specific physical, cognitive, and collaborative demands of individual therapies.

The observed relationship between Hyperthermia, Treatment-Related Burden, and Perceived Lack of Benefit suggests an important clinical interaction. Hyperthermia often demands significant physical endurance (e.g., intense heat exposure, exhaustion) and time commitment. Because its therapeutic mechanisms are systemic or radiosensitizing rather than immediately tangible, the lack of immediate subjective symptom relief—combined with high physical burden—may lead patients to question its overall efficacy. In contrast, IV Vitamin C and Mistletoe were frequently described as providing palpable, short-term energetic or psychosocial benefits (“feeling good right after”), which reinforced their Perceived Worth despite financial costs.

Furthermore, modality familiarity directly influenced the pathways to Intervention Coherence. Therapies such as Vitamin C and hyperthermia leverage common cultural/biological concepts (e.g., vitamins for immunity, fever for fighting illness), allowing patients to achieve immediate intuitive coherence. Conversely, Mistletoe therapy, which is less familiar in Western healthcare environments, demanded active Knowledge-Building and Research, where patients acted as self-advocates to establish intervention rationale.

Finally, IV Vitamin C served as a flashpoint for Gaps in Conventional-Integrative Collaboration. Because IV Vitamin C is one of the most widely recognized therapies, patients were more likely to discuss it with their conventional oncologists, frequently encountering warnings regarding antioxidant-chemotherapy interactions. This highlights that communication barriers are highest for high-profile therapies where conventional and integrative paradigms directly clash.

An interesting tension emerged regarding patient perceptions of how integrative oncology should relate to conventional cancer treatment. While the integrative oncology team and literature emphasize complementary integration with conventional medicine, participants held varying perspectives on this relationship. Most participants expressed that integrative oncology should work collaboratively with conventional approaches without replacing them, while a few articulated different expectations about the role and prominence of integrative methods. These varied perspectives suggest an important implication for clinicians: there is a clinician burden to explicitly communicate and emphasize that integrative oncology is designed to work synergistically with and not in place of conventional treatment protocols. This finding highlights the need for clear, consistent messaging from integrative practitioners to ensure that patients understand the complementary nature of these approaches. Failure to establish this clarity may lead to misaligned expectations and potentially compromise both patient safety and the credibility of integrative oncology within conventional medical settings.

This study addresses an important gap in the Canadian literature and practice on integrative naturopathic oncology. Although use of complementary and integrative therapies is acceptable and common among people with metastatic cancer, access remains uneven and often depends on private payment and self-navigation [27]. The high cost of treatments, travel expenses, and time away from family create an equity crisis, making comprehensive supportive care accessible only to those who can afford it. This shows that high acceptability alone cannot guarantee fair care. Without official policy changes and insurance coverage, the current system naturally creates inequalities, leaving lower-income metastatic cancer patients behind. Future models of integrative naturopathic oncology should therefore prioritize collaboration between oncology and integrative providers, transparent communication about treatment goals and safety, and service delivery approaches that reduce financial and logistical barriers.

Taken together, these findings suggest that integrative naturopathic oncology is valued not only for symptom management but also for the relational and meaning-making support it provides in advanced cancer. However, acceptability alone is insufficient to ensure equitable access or sustained use. In the absence of coordinated communication, financial coverage, and clear pathways for collaboration with oncology teams, patients continue to shoulder a significant navigation burden. More integrated models of care, with explicit attention to safety, communication, and affordability, may help reduce this burden and better support patients with metastatic cancer.

### Limitations

This study has several limitations. First, participants were recruited from a single specialized integrative oncology clinic in British Columbia operating in a private fee-for-service context, which may limit transferability to public cancer centres or other settings where integrative services are less available. Second, the sample of 12 participants, although adequate for qualitative descriptive analysis, may not capture the breadth of experiences among people living with metastatic cancer, particularly those from rural and remote communities, Indigenous populations, francophone groups, and other equity-deserving populations. The small sample size also restricts quantitative generalization regarding modality-specific outcomes. The heterogeneous regimens utilized by participants mean that these observed modality-specific patterns represent preliminary qualitative trends rather than statistically powered subgroup comparisons. Third, the specific modalities examined, including resource-intensive and costly interventions, may have shaped participants’ perceptions of burden and acceptability and may not reflect experiences in programs focused on lower-intensity integrative approaches. Finally, although the TFA provided a useful structure for analysis, it may not fully capture health-system, regulatory, and equity-related influences on access and legitimacy.

## 5. Conclusions

This qualitative examination of acceptability provides an important but incomplete picture of integrative naturopathic oncology’s potential role in cancer care. While acceptability is a necessary precursor to implementation, it is not sufficient on its own. Future research should prioritize rigorously designed randomized controlled trials to examine the effectiveness of integrative oncology interventions across diverse patient populations and clinical contexts. When acceptability evidence is paired with robust effectiveness data, organizations such as ASCO and provincial cancer agencies will have the evidentiary foundation needed to make informed recommendations regarding integrative oncology integration. Such paired evidence would support clinicians in confidently referring patients to integrative oncology while maintaining oversight of treatment protocols and ensuring alignment with conventional care plans. The pathway forward requires both qualitative research illuminating patient perspectives and quantitative research establishing clinical outcomes; together, these evidence streams can transform integrative oncology from a marginally accepted practice to an evidence-supported complement to conventional cancer care.

### Implications

Implications of these findings for the medical community include:

- Recognize that patients may use integrative naturopathic oncology alongside conventional treatment to address unmet physical, emotional, informational, and supportive-care needs.
- Create nonjudgmental opportunities for patients to disclose integrative therapy use and discuss potential benefits, risks, and treatment interactions.
- Improve communication and coordination between conventional oncology and qualified integrative healthcare providers to reduce fragmented care.
- Address financial barriers that may limit equitable access to supportive integrative services.
- Prioritize rigorous research evaluating the safety, effectiveness, implementation, and equity implications of integrative oncology approaches in metastatic cancer care.

## Figures and Tables

**Table 1:** Participant demographics and clinical characteristics (*n* = 12).

Variable	*n* (%)
**Sex**	
Female	10 (83%)
Male	2 (17%)
**Age range**	
18–29 years	1 (8%)
40–49 years	4 (33%)
50–59 years	4 (33%)
60–69 years	2 (17%)
70–79 years	1 (8%)
**Cancer Types**	
Colorectal Cancer	4 (33%)
Breast Cancer	3 (25%)
Lung Cancer	2 (17%)
Others	3 (25%)
**Place of Residence**	
British Columbia	10 (83%)
Outside of BC	2 (17%)
**Integrative Oncology Modalities Received**	
Naturopathic Consultations (unspecified)	7 (58%)
Hyperthermia	6 (50%)
Intravenous (IV) Therapy	4 (33%)
Mistletoe Therapy	3 (25%)
Acupuncture	3 (25%)
Other (Massage, Craniosacral Therapy, Hyperbaric Oxygen Therapy, Nutritional Therapy)	5 (42%)
**Duration of Integrative Oncology Care**	
<1 year (e.g., several months to 9 months)	2 (17%)
>1 year (e.g., 2–3 years, 3+ years)	3 (25%)
Unspecified	7 (58%)

**Table 2:** Thematic Acceptance Matrix (TFA Framework).

Theme/TFA Construct	Key Aspects	Descriptive Summary	Exemplary Quote
**Affective Attitude**	Emotional support and trust personalized, holistic care Feeling heard and valued Contrast with conventional care and systemic marginalization Disappointment Evolving perception of integrative naturopathic oncology	Participants generally described integrative naturopathic oncology as supportive, relational, and individualized. They valued having time to discuss concerns and feeling recognized as a whole person rather than solely through their diagnosis. However, some patients face frustration and disappointment due to the marginalization of integrative care within the broader cancer system. Participants also described differing trajectories in their attitudes toward integrative naturopathic oncology following their metastatic cancer diagnosis.	“It’s like talking to somebody that I trust in the integrative side, that I know cares about me… rather than somebody that’s like, I’m just a number…” Participant#8
**Burden**	Financial costs Travel and time demands Treatment intensity Physical discomfort Social, emotional and relational strain Fragmented systems of care Contested legitimacy	Although participants valued care, many described substantial out-of-pocket expenses and practical demands associated with attending appointments and sustaining treatment. Some modalities were also perceived as physically demanding or uncomfortable. Most participants experienced integrative naturopathic oncology as operating within a siloed healthcare system with limited communication and alignment between conventional and integrative providers.	“Like for me that’s the biggest thing is I just can’t afford all of the treatments.” Participant#4 “but I struggled with bringing the two sides together. Like kind of the medical oncology with the integrative oncology! they don’t talk.” Participant#7
**Ethicality**	Alignment with prior belief and lived experiences Accessible, responsive, and supportive care Integrative, naturalistic, and holistic patient-centred approaches Augmentative rather than alternative	Integrative care aligned with participants’ preferences for proactive, individualized, and whole-person approaches to cancer care. Participants valued being able to make choices consistent with their beliefs and desired role in treatment decision-making.	“I’m always interested in nutrition and superfoods and taking care of my body and fitness and health… So I’ve always worked with naturopaths… So I knew right away that I wanted, you know, something in addition to Western medicine.” Participant#8
**Intervention Coherence**	Understanding treatment rationale Information seeking Provider explanations Modality-specific familiarity & experience Understanding bodily processes	Participants generally valued understanding why therapies were recommended and how they might support their cancer care. Familiarity and perceived plausibility varied by modality; some participants required additional research or discussion to understand treatments such as mistletoe therapy.	“And then I feel like integrative doctors do a much better job explaining methods of action and why, you know, you’re doing things.” Participant#6
**Opportunity Costs**	Financial trade-offs Family and relational trade-offs Physical sacrifice	Receiving integrative care required participants to make financial, time-related, and inter-personal trade-offs. Despite these costs, many considered the perceived benefits sufficiently meaningful to justify continued participation.	“Yeah, I had to give up, I mean a lot of things like things that weren’t needs in order to pay for my care there. I had to give up all the time and I had to leave work and so things like that. I had to leave my family, my home.” Participant#1
**Perceived Effectiveness**	Symptom relief Improved energy or well-being Emotional support Enhanced sense of control Variable benefit Dynamic appraisal of effectiveness	Participants commonly described perceived improvements in well-being, energy, symptom management, emotional coping, and confidence in navigating cancer care. However, perceived benefit was not universal and varied across individuals and modalities. A few participants experienced limited or no perceived benefit early in treatment, leading to disappointment and doubt.	“I always find it more building than depleting going in for-integrative oncology.” Participant#6
**Self-Efficacy**	Confidence in following recommendations Confidence building though knowledge and guidance Treatment decision-making Active role Navigating multiple care systems From patient to advocate	Participants built their confidence in managing their integrative treatment though information and guidance, followed care recommendations, and took an active role in treatment decisions. Some participants described moving from feeling caught between conventional and integrative care toward greater agency in managing both. And for some participants, this self-efficacy evolved into active advocacy for others.	“But any questions I had were always answered and everything was explained to me before going in and nothing was forced on me either. It was all like, my choice, of course, to do any of this.” Participant#1
**Motivations for Engaging in Integrative Naturopathic Oncology (emergent theme)**	Unmet needs and uncertainty of cancer Desire for additional support and desire to live Symptom concerns Dissatisfaction with conventional-care limitations Recommendations from others	Participants sought integrative care to address unmet physical, emotional, informational, and relational needs. Motivations often reflected a desire for more time, guidance, and support than they perceived available within conventional oncology alone.	“I guess I felt kind of that I didn’t have anything to lose by doing more than what the cancer agency was offering me. Just knowing that I didn’t have a very good prognosis and just wanting to give myself as much time as I could.” Participant#2
**Cross-cutting modality-specific patterns**	Differing burden, coherence, and perceived benefit between IV vitamin C, hyperthermia, and mistletoe therapy	Acceptability was not uniform across modalities. IV vitamin C was often associated with perceived short-term benefits; hyperthermia was more frequently described as physically burdensome or of uncertain benefit; and mistletoe therapy commonly required additional explanation and personal research to establish coherence.	“Yeah, so the hyperthermia I didn’t do a whole lot of research on because I felt that it had Very minimal chance of interacting with my treatment. You know, like, in my head, it wasn’t. It didn’t raise red flags about being harmful in any way or harming my treatment. The mistletoe and the vitamin C, I definitely looked into kind of on two different fronts. One, to make sure that they didn’t interact with my medication, and two, that it didn’t have any impact on my liver failure symptoms because many natural health products, you know, have been known to have side liver. And so I wanted to make sure that nothing would be harmful for my liver either.” Participant#2

## Data Availability

The qualitative data supporting the findings of this study are not publicly available to preserve participant confidentiality and privacy within a single-clinic setting. Data may be made available by the corresponding author upon reasonable request.

## Supplementary Materials

The following supporting information can be downloaded at: https://www.mdpi.com/article/10.3390/curroncol33100609/s1, File S1: COREQ (COnsolidated criteria for REporting Qualitative research) Checklist.

## Abbreviations

The following abbreviations are used in this manuscript:

ASCO	American Society of Clinical Oncology
BC	British Columbia
CAM	Complementary and alternative medicine
CORQR	Consolidated Criteria for Reporting Qualitative Research
IV	Intravenous
NCCN	National Comprehensive Cancer Network
TFA	Theoretical Framework of Acceptability

## Appendix A. Interview Guide

### A.1. Background & Care Journey

- Can you tell me a little about your cancer journey to start?
- How long ago were you diagnosed with metastatic cancer?
- What specific cancer were you diagnosed with?
- What has that experience been like for you?
- Can you share what treatments you have received so far?

### A.2. Motivations for Seeking Integrative Naturopathic Oncology

- How did you first hear about integrative naturopathic oncology care options in general and/or the IHC clinic?
- Can you share why you ultimately decided to undergo integrative naturopathic oncology treatment?
  ○ Any specific physical, emotional, spiritual, social, informational reasons for this decision?

### A.3. Perceptions of Integrative Naturopathic Oncology

- What were your overall expectations when you first started receiving integrative naturopathic oncology care?
  ○ Would you say those expectations have been met?
  ○ How does the care you have received at this clinic compare to that you have received at other clinics (conventional or otherwise)?
- How did your relationship with your integrative naturopathic care clinician vs. your conventional oncologist shape your treatment journey?
  ○ Did you feel seen and heard by your clinician?
  ○ How would you describe the level of support you received in making complex treatment decisions?
  ○ Did you feel your clinician was nonjudgmental regarding your treatment decisions or compliance?
  ○ In what ways did your clinician’s approach (e.g., taking time, positive regard, collaborative style) influence your care experience?

### A.4. Acceptability (TFA Domains)

**Affective Attitude**

- How would you describe your overall experience with integrative naturopathic oncology?
- Are there specific things you have liked or disliked about your experiences to date?

**Burden**

- What effort (time, energy, financial, emotional) has been involved in participating in integrative naturopathic oncology?
- Have you encountered any barriers to accessing integrative naturopathic oncology?
  ○ Access, cost, communication with healthcare providers, family views)

**Ethicality**

- Would you say that it is important that the care you receive (conventional or otherwise) fits well with your overall value and belief system about health and wellness?
  ○ How well aligned do you think the treatment you have received at IHC is with your personal values and beliefs about health and overall wellness?

**Intervention Coherence**

- Can you tell me the specific treatments you have received at IHC to date?
  ○ How well do you understand the way these treatments work to address your symptoms and treat your diagnosis?
  ○ How helpful has your clinician at IHC been in explaining the treatment you receive to you?
  ○ How important is it that you understand how the treatment you receive works?
- Has this experience changed how you view or approach your cancer treatment overall?

**Opportunity Costs**

- Have you had to give up anything (time, other treatments, resources) in order to receive integrative naturopathic oncology?
  ○ Can you tell me what you had to give up and why you felt this was necessary?

**Perceived Effectiveness**

- How effective would you characterize the treatment you have received at IHC for your cancer diagnosis/associated treatment-related effects?
  ○ In what ways do you feel this care has improved your overall quality of life and well-being?
  ○ What benefits have you noticed, if any? (Probe: symptom management, emotional support, quality of life, sense of control)
- Any side effects you have noticed since receiving treatment and how were these addressed by your doctor?
- In your experience, how do you think the treatment works to create such changes?

**Self-efficacy**

- Can you describe how it has been for you to follow the treatment-related recommendations provided by your integrative naturopathic care team?
  ○ Has there been a time where you found it difficult to follow a treatment regimen or recommendation provided to you by the team?
  ○ What do you think was the reason for this (i.e., lack of support, resources, information, etc.)?

### A.5. Overall Reflections

- If given the opportunity, would you choose this care again? What factors would influence your decision?
- What advice would you give to another patient considering integrative naturopathic oncology?
- Is there anything else you would like to share about your experience that we haven’t covered?
